# The genome sequence of a beetle, *Pycnomerus fuliginosus* Erichson, 1842

**DOI:** 10.12688/wellcomeopenres.23770.1

**Published:** 2025-03-19

**Authors:** Olga Sivell, Susan C. Taylor, Maxwell V. L. Barclay, Stephanie Skipp, Michael F. Geiser, Natural History Museum Genome Acquisition Lab, Darwin Tree of Life Barcoding collective, Wellcome Sanger Institute Tree of Life Management, Samples and Laboratory team, Wellcome Sanger Institute Scientific Operations: Sequencing Operations, Wellcome Sanger Institute Tree of Life Core Informatics team, Tree of Life Core Informatics collective, Darwin Tree of Life Consortium

**Affiliations:** 1 Natural History Museum, London, England, UK; 2 Dipterists Forum, Northchurch, England, UK

**Keywords:** Pycnomerus fuliginosus, beetle, genome sequence, chromosomal, Coleoptera

## Abstract

We present a genome assembly from a female *Pycnomerus fuliginosus* (beetle; Arthropoda; Insecta; Coleoptera; Zopheridae). The genome sequence has a total length of 359.22 megabases. Most of the assembly (95.81%) is scaffolded into 11 chromosomal pseudomolecules, including the X sex chromosome. The mitochondrial genome has also been assembled and is 17.21 kilobases in length. Gene annotation of this assembly on Ensembl identified 11,547 protein-coding genes.

## Species taxonomy

Eukaryota; Opisthokonta; Metazoa; Eumetazoa; Bilateria; Protostomia; Ecdysozoa; Panarthropoda; Arthropoda; Mandibulata; Pancrustacea; Hexapoda; Insecta; Dicondylia; Pterygota; Neoptera; Endopterygota; Coleoptera; Polyphaga; Cucujiformia; Tenebrionoidea; Zopheridae; Zopherinae; Pycnomerini; *Pycnomerus*; *Pycnomerus fuliginosus* Erichson, 1842 (NCBI:txid878397)

## Background

*Pycnomerus fuliginosus* Erichson, 1842 is a species of a beetle from the family Zopheridae, commonly called ironclad beetles. This species is elongated, parallel sided, tenebrionid-like in appearance with expanded genae, reddish-brown in colour, with striatopunctate elytra with single rows of golden setae and tarsi 4-4-4. It is a small species measuring 5.5–6.0 mm ( Ivie, 2002; O’Connor *et al.*, 1983).

The tribe Pycnomerini Erichson 1845, previously in Colydiidae (Ivie, M.A.), was revised by Ślipiński and Lawrence (1999) and moved to Zopheridae. Keys to genera of Zopheridae are provided by Ślipiński and Lawrence (1999) and Ivie (2002). Zopheridae and Colydiidae have now both been downgraded to subfamilies of a broader Zopheridae (e.g. see Duff, 2018), and *Pycnomerus* is the only genus of subfamily Zopherinae occurring in Britain and Ireland.

The genus *Pycnomerus* Erichson 1842 includes 34 species worldwide, including five species occurring in Europe: *P. italicus* Ganglbauer, 1899, endemic to Italy ( Pezzi *et al.*, 2022); *P. terebrans* (Olivier, 1790); *P. inexspectus* Jacquelin du Val, 1858, found in greenhouses in a few countries; *P. angulatus* Broun, 1893 introduced to Ireland from New Zealand in recent years ( Alexander & Anderson, 2012), and *P. fuliginosus*, established in Britain and Ireland ( Iwan & Löbl, 2020). Numerous invertebrate species have been recently imported to Britain and Ireland from Australia and New Zealand (e.g. see Walters *et al.*, 2016).

*Pycnomerus fuliginosus* was accidentally introduced to Britain from Australia and was first reported in Britain in 1964. It has been suggested it was possibly brought in with horticultural commerce ( O’Connor *et al.*, 1983). Since then, it became established and has been expanding its distribution. It has been recorded mostly from southern England and Wales, with few records from the Midlands and one near Manchester ( Alexander, 2019; Allen, 1968; Morgan, 1979; Twinn & Hunter, 1967; Welch, 1964). It has been known in Ireland since 1981 ( Alexander & Anderson, 2012; O’Connor *et al.*, 1983). It is a relatively uncommon species, living under the bark of conifers and hardwood trees ( James, 2018), but adults were observed flying in sunlight around a pile of cut branches at Bookham Common Surrey (MVL Barclay, personal observation). Both larvae and adults of Pycomerini are associated with rotten plant material. According to Denux and Zagatti (2010), they are likely predatory on saproxylic insects.

Relatively little is known about the biology of *P. fuliginosus*, and many related species await description. The high-quality genome of *P. fuliginosus* presented here was sequenced from a single specimen (NHMUK014452846; SAMEA11024993) from Penryn, England. The genome was sequenced as part of the Darwin Tree of Life Project, a collaborative effort to sequence all named eukaryotic species in the Atlantic Archipelago of Britain and Ireland. It will aid research on taxonomy, phylogeny and biology of *Pycnomerus*, family Zopheridae and the superfamily Tenebrionoidea.

## Genome sequence report

### Sequencing data

**Figure 1.**
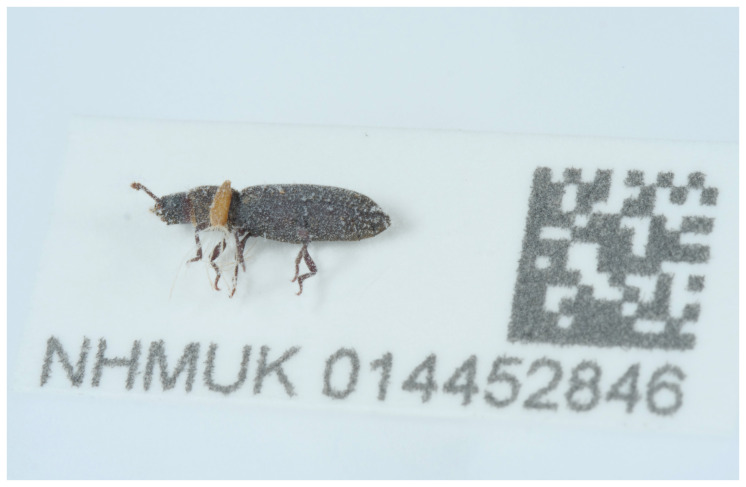
Photograph of the *Pycnomerus fuliginosus* (icPycFuli2) specimen used for genome sequencing.

The genome of a specimen of *Pycnomerus fuliginosus* ( Figure 1) was sequenced using Pacific Biosciences single-molecule HiFi long reads, generating 22.55 Gb from 1.84 million reads. GenomeScope analysis of the PacBio HiFi data estimated the haploid genome size at 365.18 Mb, with a heterozygosity of 0.71% and repeat content of 46.84%. These values provide an initial assessment of genome complexity and the challenges anticipated during assembly. Based on this estimated genome size, the sequencing data provided approximately 59.0x coverage of the genome. Chromosome conformation Hi-C sequencing produced 199.06 Gb from 1,318.25 million reads. Table 1 summarises the specimen and sequencing information, including the BioProject, study name, BioSample numbers, and sequencing data for each technology.

**Table 1.** Specimen and sequencing data for *Pycnomerus fuliginosus*.

Project information			
**Study title**	Pycnomerus fuliginosus		
**Umbrella BioProject**	PRJEB59804		
**Species**	*Pycnomerus fuliginosus*		
**BioSpecimen**	SAMEA11024993		
**NCBI taxonomy ID**	878397		
Specimen information			
**Technology**	**ToLID**	**BioSample accession**	**Organism part**
**PacBio long read sequencing**	icPycFuli2	SAMEA11025200	abdomen
**Hi-C sequencing**	icPycFuli1	SAMEA9359522	head and thorax
**RNA sequencing**	icPycFuli3	SAMEA114806053	whole organism
Sequencing information			
**Platform**	**Run accession**	**Read count**	**Base count (Gb)**
**Hi-C Illumina NovaSeq 6000**	ERR10890754	1.32e+09	199.06
**PacBio Sequel IIe**	ERR10879942	1.84e+06	22.55
**RNA Illumina NovaSeq X**	ERR13999062	8.69e+07	13.13

### Assembly statistics

The primary haplotype was assembled, and contigs corresponding to an alternate haplotype were also deposited in INSDC databases. The assembly was improved by manual curation, which corrected 14 misjoins or missing joins and removed three haplotypic duplications. These interventions reduced the total assembly length by 1.79% and decreased the scaffold count by 10.53%. The final assembly has a total length of 359.22 Mb in 50 scaffolds, with 59 gaps, and a scaffold N50 of 37.94 Mb ( Table 2).

**Table 2.** Genome assembly data for *Pycnomerus fuliginosus*.

Genome assembly		
Assembly name	icPycFuli2.1	
Assembly accession	GCA_963924575.1	
*Alternate haplotype accession*	*GCA_963924625.1*	
Assembly level for primary assembly	chromosome	
Span (Mb)	359.22	
Number of contigs	109	
Number of scaffolds	50	
Longest scaffold (Mb)	56.25	
Assembly metric	Measure	*Benchmark*
Contig N50 length	7.63 Mb	*≥ 1 Mb*
Scaffold N50 length	37.94 Mb	*= chromosome N50*
Consensus quality (QV)	Primary: 69.3; alternate: 68.5; combined 68.9	*≥ 40*
*k*-mer completeness	Primary: 83.08%; alternate: 75.60%; combined: 98.25%	*≥ 95%*
BUSCO *	C:99.3%[S:98.7%,D:0.6%], F:0.2%,M:0.5%,n:2,124	*S > 90%; D < 5%*
Percentage of assembly mapped to chromosomes	95.83%	*≥ 90%*
Sex chromosomes	X	*localised homologous pairs*
Organelles	Mitochondrial genome: 17.21 kb	*complete single alleles*
Genome annotation of assembly GCA_963924575.1 at Ensembl		
Number of protein-coding genes	11,547	
Number of non-coding genes	1,002	
Number of gene transcripts	17,565	

* BUSCO scores based on the endopterygota_odb10 BUSCO set using version 5.4.3. C = complete [S = single copy, D = duplicated], F = fragmented, M = missing, n = number of orthologues in comparison.

**Figure 2.**
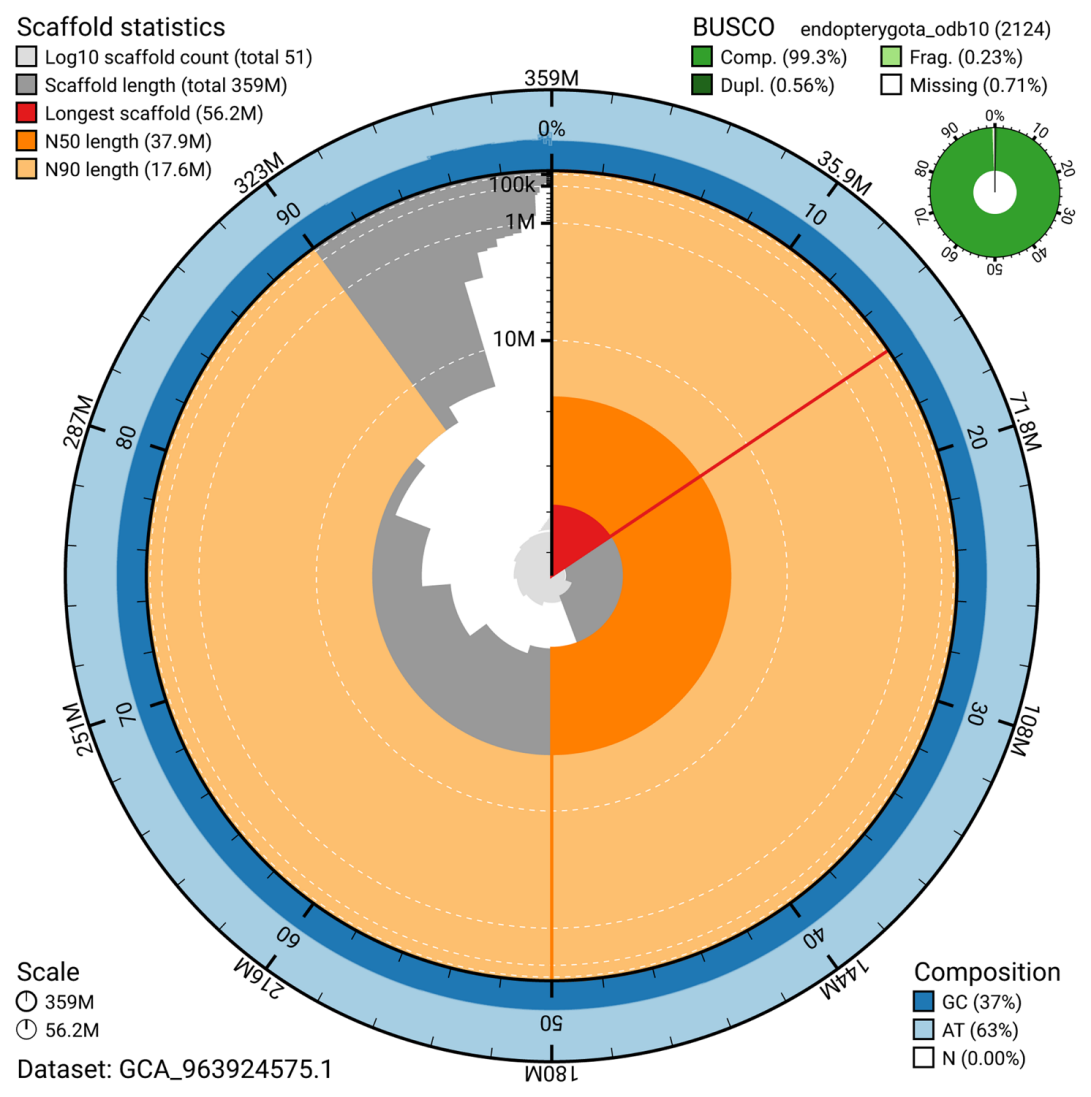
Genome assembly of *Pycnomerus fuliginosus*, icPycFuli2.1: metrics. The BlobToolKit snail plot provides an overview of assembly metrics and BUSCO gene completeness. The circumference represents the length of the whole genome sequence, and the main plot is divided into 1,000 bins around the circumference. The outermost blue tracks display the distribution of GC, AT, and N percentages across the bins. Scaffolds are arranged clockwise from longest to shortest and are depicted in dark grey. The longest scaffold is indicated by the red arc, and the deeper orange and pale orange arcs represent the N50 and N90 lengths. A light grey spiral at the centre shows the cumulative scaffold count on a logarithmic scale. A summary of complete, fragmented, duplicated, and missing BUSCO genes in the endopterygota_odb10 set is presented at the top right. An interactive version of this figure is available at https://blobtoolkit.genomehubs.org/view/GCA_963924575.1/dataset/GCA_963924575.1/snail.

**Figure 3.**
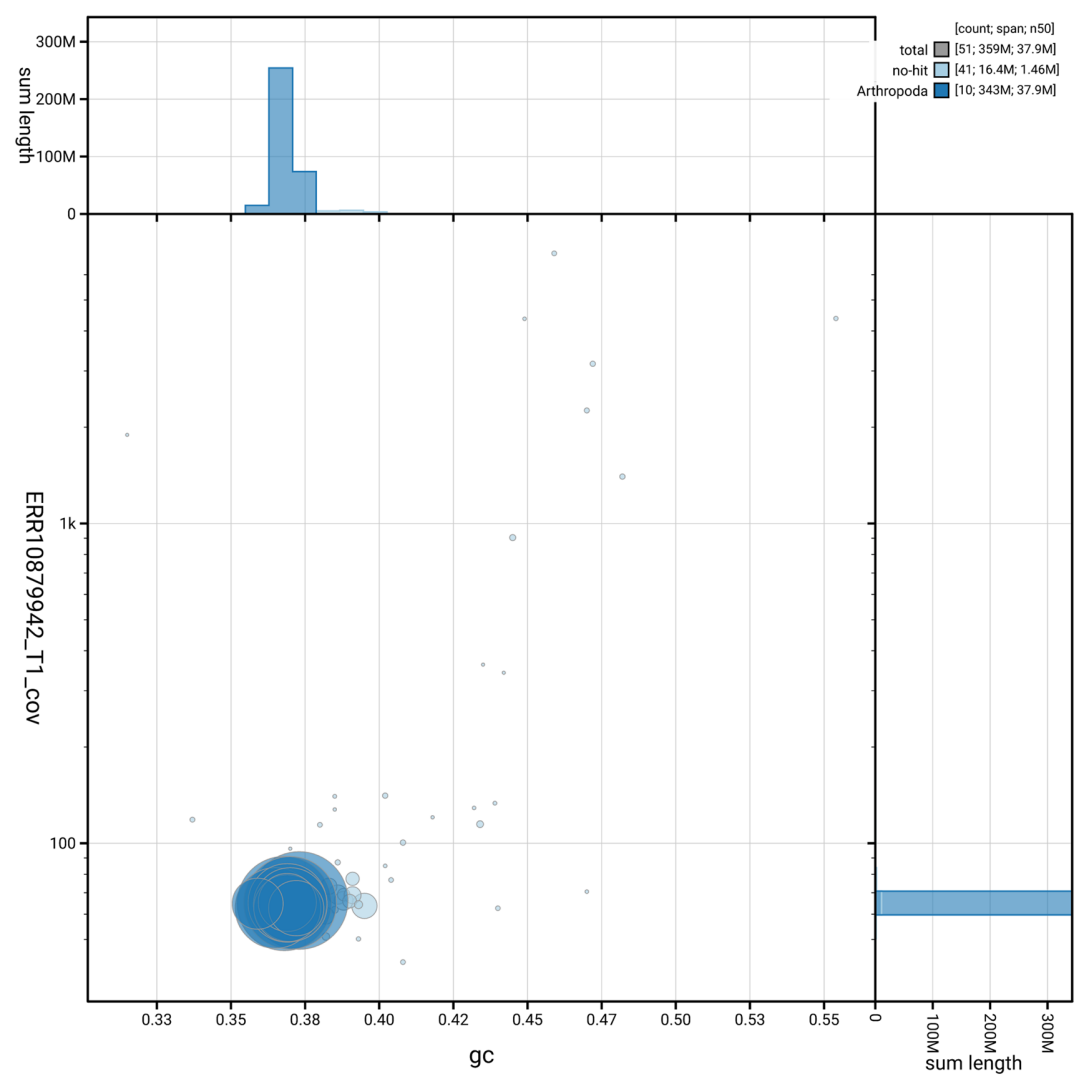
Genome assembly of *Pycnomerus fuliginosus*, icPycFuli2.1: BlobToolKit GC-coverage plot. Blob plot showing sequence coverage (vertical axis) and GC content (horizontal axis). The circles represent scaffolds, with the size proportional to scaffold length and the colour representing phylum membership. The histograms along the axes display the total length of sequences distributed across different levels of coverage and GC content. An interactive version of this figure is available at https://blobtoolkit.genomehubs.org/view/GCA_963924575.1/blob.

**Figure 4.**
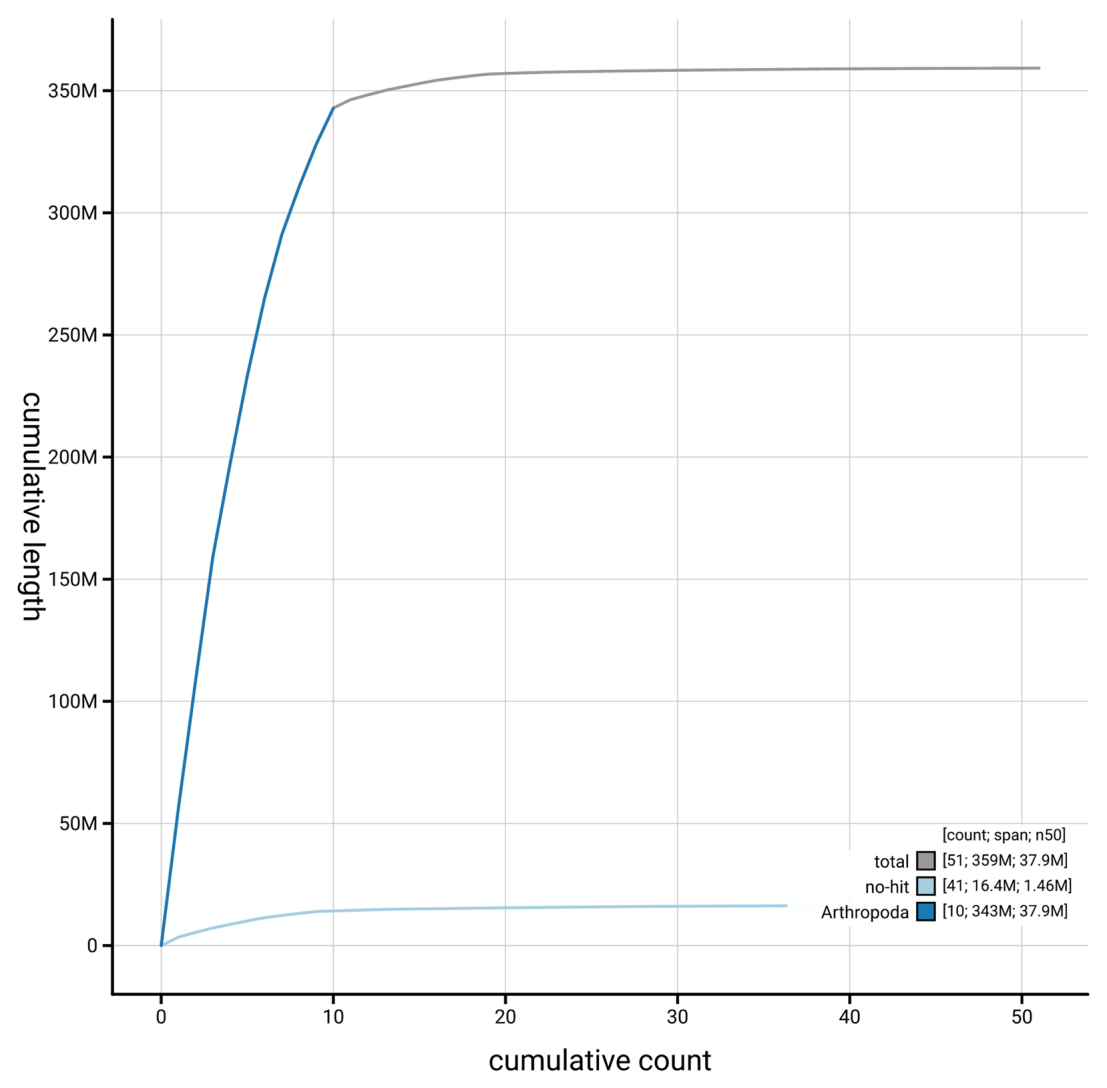
Genome assembly of *Pycnomerus fuliginosus*, icPycFuli2.1: BlobToolKit cumulative sequence plot. The grey line shows cumulative length for all scaffolds. Coloured lines show cumulative lengths of scaffolds assigned to each phylum using the buscogenes taxrule. An interactive version of this figure is available at https://blobtoolkit.genomehubs.org/view/GCA_963924575.1/dataset/GCA_963924575.1/cumulative.

The snail plot in Figure 2 provides a summary of the assembly statistics, indicating the distribution of scaffold lengths and other assembly metrics. Figure 3 shows the distribution of scaffolds by GC proportion and coverage. Figure 4 presents a cumulative assembly plot, with separate curves representing different scaffold subsets assigned to various phyla, illustrating the completeness of the assembly.

**Figure 5.**
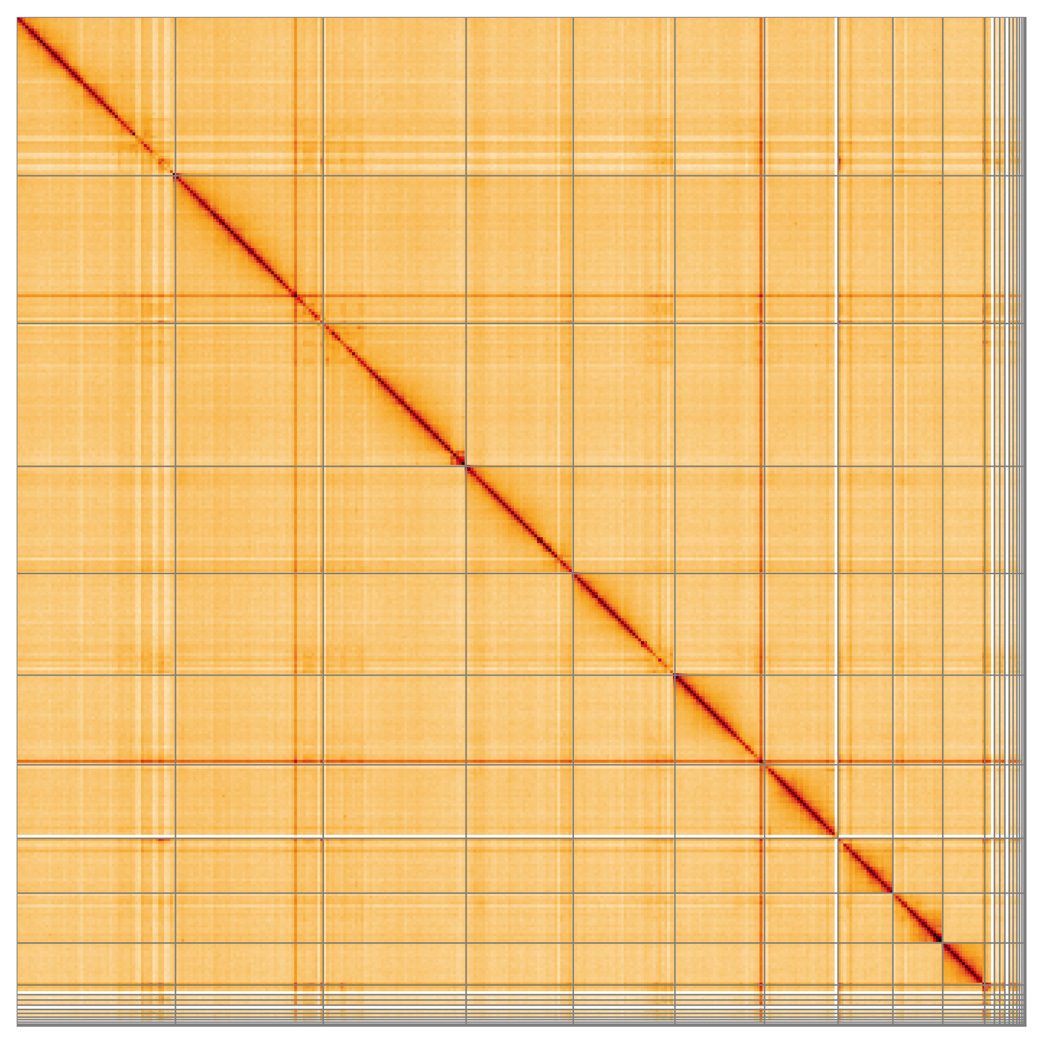
Genome assembly of *Pycnomerus fuliginosus:* Hi-C contact map of the icPycFuli2.1 assembly, visualised using HiGlass. Chromosomes are shown in order of size from left to right and top to bottom. An interactive version of this figure may be viewed at https://genome-note-higlass.tol.sanger.ac.uk/l/?d=HQQV4a_CQc6bvNKIzgJKYA.

Most of the assembly sequence (95.83%) was assigned to 11 chromosomal-level scaffolds, representing 10 autosomes and the X sex chromosome. These chromosome-level scaffolds, confirmed by Hi-C data, are named according to size ( Figure 5; Table 3). During curation, the X chromosome X was assigned by synteny to *Schizotus pectinicornis* (GCA_951805265.1) ( Lyszkowski *et al.*, 2024).

**Table 3.** Chromosomal pseudomolecules in the genome assembly of *Pycnomerus fuliginosus*, icPycFuli2.

**INSDC accession**	**Name**	**Length (Mb)**	**GC%**
OZ004617.1	1	56.25	37.5
OZ004618.1	2	52.38	37
OZ004619.1	3	50.57	37
OZ004620.1	4	37.94	36.5
OZ004621.1	5	36.06	37
OZ004622.1	6	31.7	37
OZ004623.1	7	26.06	37
OZ004624.1	8	19.45	37
OZ004625.1	9	17.57	37
OZ004627.1	10	1.33	38.5
OZ004626.1	X	14.89	36
OZ004628.1	MT	0.02	31.5

The mitochondrial genome was also assembled. This sequence is included as a contig in the multifasta file of the genome submission and as a standalone record in GenBank.

### Assembly quality metrics

The estimated Quality Value (QV) and *k*-mer completeness metrics, along with BUSCO completeness scores, were calculated for each haplotype and the combined assembly. The QV reflects the base-level accuracy of the assembly, while *k*-mer completeness indicates the proportion of expected *k*-mers identified in the assembly. BUSCO scores provide a measure of completeness based on benchmarking universal single-copy orthologues.

The primary haplotype has a QV of 69.3, and the combined primary and alternate assemblies achieve an estimated QV of 68.9. The *k*-mer completeness for the primary haplotype is 83.08%, and for the alternate haplotype it is 75.60%. The combined primary and alternate assemblies achieve a *k*-mer completeness of 98.25%. BUSCO analysis using the endopterygota_odb10 reference set ( *n* = 2,124) indicated a completeness score of 99.3% (single = 98.7%, duplicated = 0.6%).

Table 2 provides assembly metric benchmarks adapted from Rhie *et al.* (2021) and the Earth BioGenome Project (EBP) Report on Assembly Standards September 2024. The assembly achieves the EBP reference standard of **6.C.Q69**.

## Genome annotation report

The *Pycnomerus fuliginosus* genome assembly (GCA_963924575.1) was annotated at the European Bioinformatics Institute (EBI) on Ensembl Rapid Release. The resulting annotation includes 17,565 transcribed mRNAs from 11,547 protein-coding and 1,002 non-coding genes ( Table 2; https://rapid.ensembl.org/Pycnomerus_fuliginosus_GCA_963924575.1/Info/Index). The average transcript length is 9,484.21. There are 1.40 coding transcripts per gene and 4.75 exons per transcript.

## Methods

### Sample acquisition and DNA barcoding

An adult female *Pycnomerus fuliginosus* (specimen ID NHMUK014452846, ToLID icPycFuli2) was collected from University of Exeter Falmouth Campus, Penryn, England, UK (latitude 50.17, longitude –5.12) on 2021-06-29, using an aerial net. The specimen was collected by Olga Sivell (Natural History Museum), identified by Sue Taylor (Dipterists Forum) and preserved by dry freezing (–80 °C).

The specimen used for Hi-C sequencing (specimen ID NHMUK014433208, ToLID icPycFuli1) was collected from Bookham Common, Leatherhead, England, UK on 2021-04-18 by handpicking. The specimen was collected and identified by Maxwell Barclay (Natural History Museum) and preserved by dry freezing (–80 °C).

The specimen used for RNA sequencing (specimen ID NHMUK014440698, ToLID icPycFuli3) was collected from Wimbledon Common, England, United Kingdom (latitude 51.44, longitude –0.23) on 2022-03-06. The specimen was collected by Michael Geiser and Stephanie Skipp (Natural History Museum), identified by Michael Geiser and preserved by dry freezing (–80 °C).

The initial identification by morphology was verified by an additional DNA barcoding process according to the framework developed by Twyford *et al*. (2024). A small sample was dissected from the specimen and stored in ethanol, while the remaining parts were shipped on dry ice to the Wellcome Sanger Institute (WSI) ( Pereira *et al.*, 2022). The tissue was lysed, the COI marker region was amplified by PCR, and amplicons were sequenced and compared to the BOLD database, confirming the species identification ( Crowley *et al.*, 2023). Following whole genome sequence generation, the relevant DNA barcode region was also used alongside the initial barcoding data for sample tracking at the WSI ( Twyford *et al.*, 2024). The standard operating procedures for Darwin Tree of Life barcoding have been deposited on protocols.io ( Beasley *et al.*, 2023).

Metadata collection for samples adhered to the Darwin Tree of Life project standards described by Lawniczak *et al*. (2022).

### Nucleic acid extraction

The workflow for high molecular weight (HMW) DNA extraction at the Wellcome Sanger Institute (WSI) Tree of Life Core Laboratory includes a sequence of procedures: sample preparation and homogenisation, DNA extraction, fragmentation and purification. Detailed protocols are available on protocols.io ( Denton *et al.*, 2023b). The icPycFuli2 sample was prepared for DNA extraction by weighing and dissecting it on dry ice ( Jay *et al.*, 2023). Tissue from the abdomen was homogenised using a PowerMasher II tissue disruptor ( Denton *et al.*, 2023a).

HMW DNA was extracted in the WSI Scientific Operations core using the Automated MagAttract v2 protocol ( Oatley *et al.*, 2023). The DNA was sheared into an average fragment size of 12–20 kb in a Megaruptor 3 system ( Bates *et al.*, 2023). Sheared DNA was purified by solid-phase reversible immobilisation, using AMPure PB beads to eliminate shorter fragments and concentrate the DNA ( Strickland *et al.*, 2023). The concentration of the sheared and purified DNA was assessed using a Nanodrop spectrophotometer and Qubit Fluorometer using the Qubit dsDNA High Sensitivity Assay kit. Fragment size distribution was evaluated by running the sample on the FemtoPulse system.

RNA was extracted from whole organism tissue of icPycFuli3 in the Tree of Life Laboratory at the WSI using the RNA Extraction: Automated MagMax™ *mir*Vana protocol ( do Amaral *et al.*, 2023). The RNA concentration was assessed using a Nanodrop spectrophotometer and a Qubit Fluorometer using the Qubit RNA Broad-Range Assay kit. Analysis of the integrity of the RNA was done using the Agilent RNA 6000 Pico Kit and Eukaryotic Total RNA assay.

### Hi-C sample preparation

Tissue from the head and thorax of the icPycFuli1 sample was processed for Hi-C sequencing at the WSI Scientific Operations core, using the Arima-HiC v2 kit. In brief, 20–50 mg of frozen tissue (stored at –80 °C) was fixed, and the DNA crosslinked using a TC buffer with 22% formaldehyde concentration. After crosslinking, the tissue was homogenised using the Diagnocine Power Masher-II and BioMasher-II tubes and pestles. Following the Arima-HiC v2 kit manufacturer's instructions, crosslinked DNA was digested using a restriction enzyme master mix. The 5’-overhangs were filled in and labelled with biotinylated nucleotides and proximally ligated. An overnight incubation was carried out for enzymes to digest remaining proteins and for crosslinks to reverse. A clean up was performed with SPRIselect beads prior to library preparation. Additionally, the biotinylation percentage was estimated using the Qubit Fluorometer v4.0 (Thermo Fisher Scientific) and Qubit HS Assay Kit and Arima-HiC v2 QC beads.

### Library preparation and sequencing

Library preparation and sequencing were performed at the WSI Scientific Operations core.

***PacBio HiFi***

At a minimum, samples were required to have an average fragment size exceeding 8 kb and a total mass over 400 ng to proceed to the low input SMRTbell Prep Kit 3.0 protocol (Pacific Biosciences, California, USA), depending on genome size and sequencing depth required. Libraries were prepared using the SMRTbell Prep Kit 3.0 (Pacific Biosciences, California, USA) as per the manufacturer's instructions. The kit includes the reagents required for end repair/A-tailing, adapter ligation, post-ligation SMRTbell bead cleanup, and nuclease treatment. Following the manufacturer’s instructions, size selection and clean up was carried out using diluted AMPure PB beads (Pacific Biosciences, California, USA). DNA concentration was quantified using the Qubit Fluorometer v4.0 (Thermo Fisher Scientific) with Qubit 1X dsDNA HS assay kit and the final library fragment size analysis was carried out using the Agilent Femto Pulse Automated Pulsed Field CE Instrument (Agilent Technologies) and gDNA 55kb BAC analysis kit.

Samples were sequenced using the Sequel IIe system (Pacific Biosciences, California, USA). The concentration of the library loaded onto the Sequel IIe was in the range 40–135 pM. The SMRT link software, a PacBio web-based end-to-end workflow manager, was used to set-up and monitor the run, as well as perform primary and secondary analysis of the data upon completion.

***Hi-C***

For Hi-C library preparation, DNA was fragmented using the Covaris E220 sonicator (Covaris) and size selected using SPRISelect beads to 400 to 600 bp. The DNA was then enriched using the Arima-HiC v2 kit Enrichment beads. Using the NEBNext Ultra II DNA Library Prep Kit (New England Biolabs) for end repair, A-tailing, and adapter ligation. This uses a custom protocol which resembles the standard NEBNext Ultra II DNA Library Prep protocol but where library preparation occurs while DNA is bound to the Enrichment beads. For library amplification, 10 to 16 PCR cycles were required, determined by the sample biotinylation percentage. The Hi-C sequencing was performed using paired-end sequencing with a read length of 150 bp on an Illumina NovaSeq 6000 instrument.

***RNA***

Poly(A) RNA-Seq libraries were constructed using the NEB Ultra II RNA Library Prep kit, following the manufacturer’s instructions. RNA sequencing was performed on the Illumina NovaSeq X instrument.

### Genome assembly, curation and evaluation

***Assembly***

Prior to assembly of the PacBio HiFi reads, a database of *k*-mer counts ( *k* = 31) was generated from the filtered reads using FastK. GenomeScope2 ( Ranallo-Benavidez *et al.*, 2020) was used to analyse the *k*-mer frequency distributions, providing estimates of genome size, heterozygosity, and repeat content.

The HiFi reads were first assembled using Hifiasm ( Cheng *et al.*, 2021) with the --primary option. Haplotypic duplications were identified and removed using purge_dups ( Guan *et al.*, 2020). The Hi-C reads were mapped to the primary contigs using bwa-mem2 ( Vasimuddin *et al.*, 2019). The contigs were further scaffolded using the provided Hi-C data ( Rao *et al.*, 2014) in YaHS ( Zhou *et al.*, 2023) using the --break option for handling potential misassemblies. The scaffolded assemblies were evaluated using Gfastats ( Formenti *et al.*, 2022), BUSCO ( Manni *et al.*, 2021) and MERQURY.FK ( Rhie *et al.*, 2020).

The mitochondrial genome was assembled using MitoHiFi ( Uliano-Silva *et al.*, 2023), which runs MitoFinder ( Allio *et al.*, 2020) and uses these annotations to select the final mitochondrial contig and to ensure the general quality of the sequence.

***Assembly curation***

The assembly was decontaminated using the Assembly Screen for Cobionts and Contaminants (ASCC) pipeline (article in preparation). Flat files and maps used in curation were generated in TreeVal ( Pointon *et al.*, 2023). Manual curation was primarily conducted using PretextView ( Harry, 2022), with additional insights provided by JBrowse2 ( Diesh *et al.*, 2023) and HiGlass ( Kerpedjiev *et al.*, 2018). Scaffolds were visually inspected and corrected as described by Howe *et al*. (2021). Any identified contamination, missed joins, and mis-joins were corrected, and duplicate sequences were tagged and removed. The sex chromosome was assigned by synteny analysis. The curation process is documented at https://gitlab.com/wtsi-grit/rapid-curation (article in preparation).

***Assembly quality assessment***

The Merqury.FK tool ( Rhie *et al.*, 2020), run in a Singularity container ( Kurtzer *et al.*, 2017), was used to evaluate *k*-mer completeness and assembly quality for the primary and alternate haplotypes using the *k*-mer databases ( *k* = 31) that were computed prior to genome assembly. The analysis outputs included assembly QV scores and completeness statistics.

A Hi-C contact map was produced for the final version of the assembly. The Hi-C reads were aligned using bwa-mem2 ( Vasimuddin *et al.*, 2019) and the alignment files were combined using SAMtools ( Danecek *et al.*, 2021). The Hi-C alignments were converted into a contact map using BEDTools ( Quinlan & Hall, 2010) and the Cooler tool suite ( Abdennur & Mirny, 2020). The contact map was visualised in HiGlass ( Kerpedjiev *et al.*, 2018).

The blobtoolkit pipeline is a Nextflow port of the previous Snakemake Blobtoolkit pipeline ( Challis *et al.*, 2020). It aligns the PacBio reads in SAMtools and minimap2 ( Li, 2018) and generates coverage tracks for regions of fixed size. In parallel, it queries the GoaT database ( Challis *et al.*, 2023) to identify all matching BUSCO lineages to run BUSCO ( Manni *et al.*, 2021). For the three domain-level BUSCO lineages, the pipeline aligns the BUSCO genes to the UniProt Reference Proteomes database ( Bateman *et al.*, 2023) with DIAMOND blastp ( Buchfink *et al.*, 2021). The genome is also divided into chunks according to the density of the BUSCO genes from the closest taxonomic lineage, and each chunk is aligned to the UniProt Reference Proteomes database using DIAMOND blastx. Genome sequences without a hit are chunked using seqtk and aligned to the NT database with blastn ( Altschul *et al.*, 1990). The blobtools suite combines all these outputs into a blobdir for visualisation.

The blobtoolkit pipeline was developed using nf-core tooling ( Ewels *et al.*, 2020) and MultiQC ( Ewels *et al.*, 2016), relying on the Conda package manager, the Bioconda initiative ( Grüning *et al.*, 2018), the Biocontainers infrastructure ( da Veiga Leprevost *et al.*, 2017), as well as the Docker ( Merkel, 2014) and Singularity ( Kurtzer *et al.*, 2017) containerisation solutions.

Table 4 contains a list of relevant software tool versions and sources.

**Table 4.** Software tools: versions and sources.

**Software tool**	**Version**	**Source**
BEDTools	2.30.0	https://github.com/arq5x/bedtools2
BLAST	2.14.0	ftp://ftp.ncbi.nlm.nih.gov/blast/executables/blast+/
BlobToolKit	4.3.9	https://github.com/blobtoolkit/blobtoolkit
BUSCO	5.5.0	https://gitlab.com/ezlab/busco
bwa-mem2	2.2.1	https://github.com/bwa-mem2/bwa-mem2
Cooler	0.8.11	https://github.com/open2c/cooler
DIAMOND	2.1.8	https://github.com/bbuchfink/diamond
fasta_windows	0.2.4	https://github.com/tolkit/fasta_windows
FastK	427104ea91c78c3b8b8b49f1a7d6bbeaa869ba1c	https://github.com/thegenemyers/FASTK
Gfastats	1.3.6	https://github.com/vgl-hub/gfastats
GoaT CLI	0.2.5	https://github.com/genomehubs/goat-cli
Hifiasm	0.16.1-r375	https://github.com/chhylp123/hifiasm
HiGlass	44086069ee7d4d3f6f3f0012569789ec138f42b84aa44357826c0b6753eb28de	https://github.com/higlass/higlass
MerquryFK	d00d98157618f4e8d1a9190026b19b471055b22e	https://github.com/thegenemyers/MERQURY.FK
Minimap2	2.24-r1122	https://github.com/lh3/minimap2
MitoHiFi	2	https://github.com/marcelauliano/MitoHiFi
MultiQC	1.14, 1.17, and 1.18	https://github.com/MultiQC/MultiQC
NCBI Datasets	15.12.0	https://github.com/nextflow-io/nextflow
Nextflow	23.04.1	https://github.com/nextflow-io/nextflow
PretextView	0.2.5	https://github.com/sanger-tol/PretextView
purge_dups	1.2.3	https://github.com/dfguan/purge_dups
samtools	1.19.2	https://github.com/samtools/samtools
sanger-tol/ascc	-	https://github.com/sanger-tol/ascc
sanger-tol/blobtoolkit	0.5.1	https://github.com/sanger-tol/blobtoolkit
Seqtk	1.3	https://github.com/lh3/seqtk
Singularity	3.9.0	https://github.com/sylabs/singularity
TreeVal	1.2.0	https://github.com/sanger-tol/treeval
YaHS	1.2a	https://github.com/c-zhou/yahs

### Genome annotation

The Ensembl Genebuild annotation system ( Aken *et al.*, 2016) was used to generate annotation for the *Pycnomerus fuliginosus* assembly (GCA_963924575.1) in Ensembl Rapid Release at the EBI. Annotation was created primarily through alignment of transcriptomic data to the genome, with gap filling via protein-to-genome alignments of a select set of proteins from UniProt ( UniProt Consortium, 2019).

### Wellcome Sanger Institute – Legal and Governance

The materials that have contributed to this genome note have been supplied by a Darwin Tree of Life Partner. The submission of materials by a Darwin Tree of Life Partner is subject to the **‘Darwin Tree of Life Project Sampling Code of Practice’**, which can be found in full on the Darwin Tree of Life website here. By agreeing with and signing up to the Sampling Code of Practice, the Darwin Tree of Life Partner agrees they will meet the legal and ethical requirements and standards set out within this document in respect of all samples acquired for, and supplied to, the Darwin Tree of Life Project.

Further, the Wellcome Sanger Institute employs a process whereby due diligence is carried out proportionate to the nature of the materials themselves, and the circumstances under which they have been/are to be collected and provided for use. The purpose of this is to address and mitigate any potential legal and/or ethical implications of receipt and use of the materials as part of the research project, and to ensure that in doing so we align with best practice wherever possible. The overarching areas of consideration are:

•     Ethical review of provenance and sourcing of the material

•     Legality of collection, transfer and use (national and international)

Each transfer of samples is further undertaken according to a Research Collaboration Agreement or Material Transfer Agreement entered into by the Darwin Tree of Life Partner, Genome Research Limited (operating as the Wellcome Sanger Institute), and in some circumstances other Darwin Tree of Life collaborators.

## Data Availability

European Nucleotide Archive: Pycnomerus fuliginosus. Accession number PRJEB59804; https://identifiers.org/ena.embl/PRJEB59804. The genome sequence is released openly for reuse. The *Pycnomerus fuliginosus* genome sequencing initiative is part of the Darwin Tree of Life (DToL) project. All raw sequence data and the assembly have been deposited in INSDC databases. Raw data and assembly accession identifiers are reported in Table 1 and Table 2.

## Author information

Members of the Natural History Museum Genome Acquisition Lab are listed here: https://doi.org/10.5281/zenodo.12159242.

Members of the Darwin Tree of Life Barcoding collective are listed here: https://doi.org/10.5281/zenodo.12158331.

Members of the Wellcome Sanger Institute Tree of Life Management, Samples and Laboratory team are listed here: https://doi.org/10.5281/zenodo.12162482.

Members of Wellcome Sanger Institute Scientific Operations: Sequencing Operations are listed here: https://doi.org/10.5281/zenodo.12165051.

Members of the Wellcome Sanger Institute Tree of Life Core Informatics team are listed here: https://doi.org/10.5281/zenodo.12160324.

Members of the Tree of Life Core Informatics collective are listed here: https://doi.org/10.5281/zenodo.12205391.

Members of the Darwin Tree of Life Consortium are listed here: https://doi.org/10.5281/zenodo.4783558.

## References

[ref-1] Abdennur N Mirny LA : Cooler: scalable storage for Hi-C data and other genomically labeled arrays. *Bioinformatics.* 2020;36(1):311–316. 10.1093/bioinformatics/btz540 31290943 PMC8205516

[ref-62] Aken BL Ayling S Barrell D : The Ensembl gene annotation system. *Database (Oxford).* 2016;2016: baw093. 10.1093/database/baw093 27337980 PMC4919035

[ref-100] Alexander KN : Saproxylic invertebrate survey of Wye Valley Woodlands Special Area of Conservation (SAC) in 2017. 2019. Reference Source

[ref-101] Alexander KNA Anderson R : The beetles of decaying wood in Ireland: a provisional annotated checklist of saproxylic Coleoptera. 2012. Reference Source

[ref-200] Allen A : A note on *Pycnomerus fuliginosus* Er.(Colydiidae) in Epping forest, Essex. *Entomologist’s Monthly Magazine.* 1968;104:160.

[ref-2] Allio R Schomaker-Bastos A Romiguier J : MitoFinder: efficient automated large-scale extraction of mitogenomic data in target enrichment phylogenomics. *Mol Ecol Resour.* 2020;20(4):892–905. 10.1111/1755-0998.13160 32243090 PMC7497042

[ref-3] Altschul SF Gish W Miller W : Basic local alignment search tool. *J Mol Biol.* 1990;215(3):403–410. 10.1016/S0022-2836(05)80360-2 2231712

[ref-4] Bateman A Martin MJ Orchard S : UniProt: the universal protein knowledgebase in 2023. *Nucleic Acids Res.* 2023;51(D1):D523–D531. 10.1093/nar/gkac1052 36408920 PMC9825514

[ref-5] Bates A Clayton-Lucey I Howard C : Sanger Tree of Life HMW DNA fragmentation: diagenode Megaruptor ^®^3 for LI PacBio. *protocols.io.* 2023. 10.17504/protocols.io.81wgbxzq3lpk/v1

[ref-6] Beasley J Uhl R Forrest LL : DNA barcoding SOPs for the Darwin Tree of Life project. *protocols.io.* 2023; [Accessed 25 June 2024]. 10.17504/protocols.io.261ged91jv47/v1

[ref-7] Buchfink B Reuter K Drost HG : Sensitive protein alignments at Tree-of-Life scale using DIAMOND. *Nat Methods.* 2021;18(4):366–368. 10.1038/s41592-021-01101-x 33828273 PMC8026399

[ref-8] Challis R Kumar S Sotero-Caio C : Genomes on a Tree (GoaT): a versatile, scalable search engine for genomic and sequencing project metadata across the eukaryotic Tree of Life [version 1; peer review: 2 approved]. *Wellcome Open Res.* 2023;8:24. 10.12688/wellcomeopenres.18658.1 36864925 PMC9971660

[ref-9] Challis R Richards E Rajan J : BlobToolKit – interactive quality assessment of genome assemblies. *G3 (Bethesda).* 2020;10(4):1361–1374. 10.1534/g3.119.400908 32071071 PMC7144090

[ref-10] Cheng H Concepcion GT Feng X : Haplotype-resolved *de novo* assembly using phased assembly graphs with hifiasm. *Nat Methods.* 2021;18(2):170–175. 10.1038/s41592-020-01056-5 33526886 PMC7961889

[ref-11] Crowley L Allen H Barnes I : A sampling strategy for genome sequencing the British terrestrial arthropod fauna [version 1; peer review: 2 approved]. *Wellcome Open Res.* 2023;8:123. 10.12688/wellcomeopenres.18925.1 37408610 PMC10318377

[ref-12] da Veiga Leprevost F Grüning BA Alves Aflitos S : BioContainers: an open-source and community-driven framework for software standardization. *Bioinformatics.* 2017;33(16):2580–2582. 10.1093/bioinformatics/btx192 28379341 PMC5870671

[ref-13] Danecek P Bonfield JK Liddle J : Twelve years of SAMtools and BCFtools. *GigaScience.* 2021;10(2): giab008. 10.1093/gigascience/giab008 33590861 PMC7931819

[ref-14] Denton A Oatley G Cornwell C : Sanger Tree of Life sample homogenisation: PowerMash. *protocols.io.* 2023a. 10.17504/protocols.io.5qpvo3r19v4o/v1

[ref-15] Denton A Yatsenko H Jay J : Sanger Tree of Life wet laboratory protocol collection V.1. *protocols.io.* 2023b. 10.17504/protocols.io.8epv5xxy6g1b/v1

[ref-440] Denux O Zagatti P : Coleoptera families other than cerambycidae, curculionidae sensu lato, chrysomelidae sensu lato and coccinellidae. *BioRisk.* 2010;4:315–406. 10.3897/biorisk.4.61

[ref-44] Diesh C Stevens GJ Xie P : JBrowse 2: a modular genome browser with views of synteny and structural variation. *Genome Biol.* 2023;24(1): 74. 10.1186/s13059-023-02914-z 37069644 PMC10108523

[ref-150] do Amaral RJV Bates A Denton A : Sanger Tree of Life RNA extraction: automated MagMax ^TM^ mirVana. *protocols.io.* 2023. 10.17504/protocols.io.6qpvr36n3vmk/v1

[ref-151] Duff AG : Checklist of beetles of the British Isles. 3rd ed. Iver: Pemberley Books,2018. 10.17504/protocols.io.8epv5xxy6g1b/v1

[ref-18] Ewels P Magnusson M Lundin S : MultiQC: summarize analysis results for multiple tools and samples in a single report. *Bioinformatics.* 2016;32(19):3047–3048. 10.1093/bioinformatics/btw354 27312411 PMC5039924

[ref-68] Ewels PA Peltzer A Fillinger S : The nf-core framework for community-curated bioinformatics pipelines. *Nat Biotechnol.* 2020;38(3):276–278. 10.1038/s41587-020-0439-x 32055031

[ref-19] Formenti G Abueg L Brajuka A : Gfastats: conversion, evaluation and manipulation of genome sequences using assembly graphs. *Bioinformatics.* 2022;38(17):4214–4216. 10.1093/bioinformatics/btac460 35799367 PMC9438950

[ref-24] Grüning B Dale R Sjödin A : Bioconda: sustainable and comprehensive software distribution for the life sciences. *Nat Methods.* 2018;15(7):475–476. 10.1038/s41592-018-0046-7 29967506 PMC11070151

[ref-25] Guan D McCarthy SA Wood J : Identifying and removing haplotypic duplication in primary genome assemblies. *Bioinformatics.* 2020;36(9):2896–2898. 10.1093/bioinformatics/btaa025 31971576 PMC7203741

[ref-26] Harry E : PretextView (Paired REad TEXTure Viewer): a desktop application for viewing pretext contact maps. 2022. Reference Source

[ref-28] Howe K Chow W Collins J : Significantly improving the quality of genome assemblies through curation. *GigaScience.* 2021;10(1): giaa153. 10.1093/gigascience/giaa153 33420778 PMC7794651

[ref-280] Ivie MA : Zopheridae Solier 1834. In: Arnett R. H., Jr., Thomas, M. C., Skelley, P. E., and Frank, J. H. (eds.). *American beetles. volume 2. Polyphaga: scarabaeoidea through curculionoidea*. CRC Press,2002;457–462. Reference Source

[ref-281] Iwan D Löbl I : Catalogue of palaearctic coleoptera. Volume 5. tenebrionoidea, revised and updated.2nd ed. Leiden, The Netherlands: Brill,2020. Reference Source

[ref-282] James TJ : Beetles of hertfordshire. Hertfordshire Natural History Society,2018. Reference Source

[ref-29] Jay J Yatsenko H Narváez-Gómez JP : Sanger Tree of Life sample preparation: triage and dissection. *protocols.io.* 2023. 10.17504/protocols.io.x54v9prmqg3e/v1

[ref-32] Kerpedjiev P Abdennur N Lekschas F : HiGlass: web-based visual exploration and analysis of genome interaction maps. *Genome Biol.* 2018;19(1): 125. 10.1186/s13059-018-1486-1 30143029 PMC6109259

[ref-33] Kurtzer GM Sochat V Bauer MW : Singularity: scientific containers for mobility of compute. *PLoS One.* 2017;12(5): e0177459. 10.1371/journal.pone.0177459 28494014 PMC5426675

[ref-34] Lawniczak MKN Davey RP Rajan J : Specimen and sample metadata standards for biodiversity genomics: a proposal from the Darwin Tree of Life project [version 1; peer review: 2 approved with reservations]. *Wellcome Open Res.* 2022;7:187. 10.12688/wellcomeopenres.17605.1

[ref-35] Li H : Minimap2: pairwise alignment for nucleotide sequences. *Bioinformatics.* 2018;34(18):3094–3100. 10.1093/bioinformatics/bty191 29750242 PMC6137996

[ref-340] Lyszkowski R Telnov D Barclay MV : The genome sequence of the Scarce Cardinal Beetle, *Schizotus pectinicornis* (Linnaeus, 1758) [version 1; peer review: 2 approved]. *Wellcome Open Res.* 2024;9:501. 10.12688/wellcomeopenres.22888.1 39640369 PMC11617827

[ref-36] Manni M Berkeley MR Seppey M : BUSCO update: novel and streamlined workflows along with broader and deeper phylogenetic coverage for scoring of eukaryotic, prokaryotic, and viral genomes. *Mol Biol Evol.* 2021;38(10):4647–4654. 10.1093/molbev/msab199 34320186 PMC8476166

[ref-37] Merkel D : Docker: lightweight Linux containers for consistent development and deployment. *Linux J.* 2014;2014(239): 2, [Accessed 2 April 2024]. Reference Source

[ref-38] Morgan MJ : *Pycnomerus fuliginosus* Er.(Col., Colydiidae) in Devon. *Entomologist’s Monthly Magazine.* 1979;114:166.

[ref-39] Oatley G Denton A Howard C : Sanger Tree of Life HMW DNA extraction: automated MagAttract v.2. *protocols.io.* 2023. 10.17504/protocols.io.kxygx3y4dg8j/v1

[ref-380] O’Connor JP Nash R Anderson R : Insects imported into Ireland. 4. Records of Dictyoptera, Hemiptera, and Coleoptera (Including *Pycnomerus fuliginosus* Erichson). *Ir Nat J.* 1983;81–83. Reference Source

[ref-40] Pereira L Sivell O Sivess L : DToL Taxon-specific standard operating Procedure for the terrestrial and freshwater arthropods working group. 2022. 10.17504/protocols.io.261gennyog47/v1

[ref-420] Pezzi M Carlomagno F Mendicino F : *Pycnomerus italicus* (Coleoptera: Zopheridae), an endemic endangered species: a new report on its presence in southern Italy. *Forests.* 2022;13(11): 1838. 10.3390/f13111838

[ref-41] Pointon DL Eagles W Sims Y : sanger-tol/treeval v1.0.0 – Ancient Atlantis. 2023. 10.5281/zenodo.10047654

[ref-47] Quinlan AR Hall IM : BEDTools: a flexible suite of utilities for comparing genomic features. *Bioinformatics.* 2010;26(6):841–842. 10.1093/bioinformatics/btq033 20110278 PMC2832824

[ref-48] Ranallo-Benavidez TR Jaron KS Schatz MC : GenomeScope 2.0 and Smudgeplot for reference-free profiling of polyploid genomes. *Nat Commun.* 2020;11(1): 1432. 10.1038/s41467-020-14998-3 32188846 PMC7080791

[ref-56] Rao SSP Huntley MH Durand NC : A 3D map of the human genome at kilobase resolution reveals principles of chromatin looping. *Cell.* 2014;159(7):1665–1680. 10.1016/j.cell.2014.11.021 25497547 PMC5635824

[ref-49] Rhie A McCarthy SA Fedrigo O : Towards complete and error-free genome assemblies of all vertebrate species. *Nature.* 2021;592(7856):737–746. 10.1038/s41586-021-03451-0 33911273 PMC8081667

[ref-50] Rhie A Walenz BP Koren S : Merqury: reference-free quality, completeness, and phasing assessment for genome assemblies. *Genome Biol.* 2020;21(1): 245. 10.1186/s13059-020-02134-9 32928274 PMC7488777

[ref-550] Ślipiński SA Lawrence JF : Phylogeny and classification of Zopheridae *sensu novo* (Coleoptera: Tenebrionoidea) with a review of the genera of Zopherinae (excluding Monommatini). *Ann Zool.* 1999;49(1–2):1–53. Reference Source

[ref-55] Strickland M Cornwell C Howard C : Sanger Tree of Life fragmented DNA clean up: manual SPRI. *protocols.io.* 2023. 10.17504/protocols.io.kxygx3y1dg8j/v1

[ref-501] Twinn DC Hunter F : A further British record of *Pycnomerus fuliginosus* Er. *Entomologist’s Monthly Magazine.* 1967;102:144.

[ref-51] Twyford AD Beasley J Barnes I : A DNA barcoding framework for taxonomic verification in the Darwin Tree of Life project [version 1; peer review: 2 approved]. *Wellcome Open Res.* 2024;9:339. 10.12688/wellcomeopenres.21143.1 39386966 PMC11462125

[ref-52] Uliano-Silva M Ferreira JGRN Krasheninnikova K : MitoHiFi: a python pipeline for mitochondrial genome assembly from PacBio high fidelity reads. *BMC Bioinformatics.* 2023;24(1): 288. 10.1186/s12859-023-05385-y 37464285 PMC10354987

[ref-63] UniProt Consortium UniProt: a worldwide hub of protein knowledge. *Nucleic Acids Res.* 2019;47(D1):D506–D515. 10.1093/nar/gky1049 30395287 PMC6323992

[ref-53] Vasimuddin M Misra S Li H : Efficient architecture-aware acceleration of BWA-MEM for multicore systems.In: *2019 IEEE International Parallel and Distributed Processing Symposium (IPDPS).*IEEE,2019;314–324. 10.1109/IPDPS.2019.00041

[ref-54] Walters JM Barclay MVL Geiser MF : *Xylotoles griseus* (Fabricius, 1775) (Cerambycidae; Lamiinae), the New Zealand fig longhorn, breeding in Devon, new to Britain and Europe. *The Coleopterist.* 2016;25(1):53–56. Reference Source

[ref-60] Welch RC : *Pycnomerus fuliginosus* Er. Col., Colydiidae) new to Britain. *Entomologist’s Monthly Magazine.* 1964;100:57–60.

[ref-61] Zhou C McCarthy SA Durbin R : YaHS: yet another Hi-C scaffolding tool. *Bioinformatics.* 2023;39(1): btac808. 10.1093/bioinformatics/btac808 36525368 PMC9848053

