# Supplementary material for: The genome sequence of a beetle, Pycnomerus fuliginosus Erichson, 1842
Source: Wellcome Open Res. 2025 Mar 19;10:144. [Version 1] doi: 10.12688/wellcomeopenres.23770.1 (PMC12006784; doi:10.12688/wellcomeopenres.23770.1)
Supplement: Peer review history

# Reviewer report: Reviewer response for version 1

**DOI:** 10.21956/wellcomeopenres.26218.r121178

**Published:** 2025-04-17

**Authors:** Chenyang Cai

**Affiliations:** 1 Chinese Academy of Sciences, Nanjing, China

The authors present a high-quality genome assembly of *Pycnomerus fuliginosus. *Most of the assembly (95.81%) is scaffolded into 11 chromosomal pseudomolecules. They also provide the mitochondrial genome of 17.21 kilobases in length. 

 I have only one question:

 The BUSCO is recently updated; which version was used in this study? 

 endopterygota_odb10.2024-01-08.tar.gz              04-Mar-2025 01:01    144M

 endopterygota_odb12.2025-04-11.tar.gz              11-Apr-2025 21:33    326M

 version 4 or version 5?

Are sufficient details of methods and materials provided to allow replication by others?

Yes

Is the rationale for creating the dataset(s) clearly described?

Yes

Are the datasets clearly presented in a useable and accessible format?

Yes

Are the protocols appropriate and is the work technically sound?

Yes

Reviewer Expertise:

beetles, phylogenomics

I confirm that I have read this submission and believe that I have an appropriate level of expertise to confirm that it is of an acceptable scientific standard.

# Reviewer report: Reviewer response for version 1

**DOI:** 10.21956/wellcomeopenres.26218.r121184

**Published:** 2025-03-28

**Authors:** Lapo Ragionieri

**Affiliations:** 1 Free University of Bolzano, Bolzano, Italy

The data note, titled “The genome sequence of a beetle, *Pycnomerus fuliginosus* Erichson, 1842” authored by Olga Sivell *et al.*, details the sequencing and genome assembly of *Pycnomerus fuliginosus*. The authors utilized long-read sequencing technology (PacBio) to generate an initial assembly, which was then scaffolded using Hi-C, resulting in a chromosome-level genome assembly. Nearly all scaffolds were assigned to chromosomes, and the assembly also includes the mitochondrial genome. The genome assembly spans a total length of 359 Mb across 11 chromosomes.

 The introduction and presentation of the results are well-documented; however, the authors did not provide information regarding the masking of the genome.

**Below are specific comments and suggestions:**

- **Title: **Zopheridae is a beetle family within Tenebrionoidea comprising less than 200 genera distributed worldwide. Within the Tenebrionoidea, it is significant as it is closely related to the Tenebrionidae, the seventh largest family in Coleoptera. It is suggested to include this information in the title (Coleoptera: Zopheridae). Recent papers addressing phylogeny in Tenebrionidae species globally and from South America use also Zopheridae as the outgroup.
- **Keywords: **should be different from title.
- **Annotation**: The annotated genome identified around 17,500 transcripts, but the genome was not masked. As the overall number of identified transcripts is smaller than what has been reported in other beetles, quantifying transposable elements within the genome might help increase the level of annotation.
- **Command Lines**: Providing the command lines used for all software applications is standard practice. Including this information would benefit readers significantly.

Are sufficient details of methods and materials provided to allow replication by others?

Yes

Is the rationale for creating the dataset(s) clearly described?

Yes

Are the datasets clearly presented in a useable and accessible format?

Yes

Are the protocols appropriate and is the work technically sound?

Yes

Reviewer Expertise:

genomics transcriptomics and proteomics

I confirm that I have read this submission and believe that I have an appropriate level of expertise to confirm that it is of an acceptable scientific standard.

## References

[1] : Phylogenomics of darkling beetles (Coleoptera: Tenebrionidae) from the Atacama Desert. *PeerJ* .2023;11: 10.7717/peerj.14848 e14848 10.7717/peerj.14848 36855434 PMC9968461

[2] : Higher level molecular phylogeny of darkling beetles (C oleoptera:T enebrionidae). *Systematic Entomology* .2014;39(3) : 10.1111/syen.12065 486-499 10.1111/syen.12065
